# Next‐generation biological control: the need for integrating genetics and genomics

**DOI:** 10.1111/brv.12641

**Published:** 2020-08-14

**Authors:** Kelley Leung, Erica Ras, Kim B. Ferguson, Simone Ariëns, Dirk Babendreier, Piter Bijma, Kostas Bourtzis, Jacques Brodeur, Margreet A. Bruins, Alejandra Centurión, Sophie R. Chattington, Milena Chinchilla‐Ramírez, Marcel Dicke, Nina E. Fatouros, Joel González‐Cabrera, Thomas V. M. Groot, Tim Haye, Markus Knapp, Panagiota Koskinioti, Sophie Le Hesran, Manolis Lyrakis, Angeliki Paspati, Meritxell Pérez‐Hedo, Wouter N. Plouvier, Christian Schlötterer, Judith M. Stahl, Andra Thiel, Alberto Urbaneja, Louis van de Zande, Eveline C. Verhulst, Louise E. M. Vet, Sander Visser, John H. Werren, Shuwen Xia, Bas J. Zwaan, Sara Magalhães, Leo W. Beukeboom, Bart A. Pannebakker

**Affiliations:** ^1^ Groningen Institute for Evolutionary Life Sciences University of Groningen PO Box 11103 9700 CC Groningen The Netherlands; ^2^ Insect Pest Control Laboratory, Joint FAO/IAEA Division of Nuclear Techniques in Food and Agriculture Vienna International Centre P.O. Box 100 1400 Vienna Austria; ^3^ Laboratory of Genetics Wageningen University & Research Droevendaalsesteeg 1 6708 PB Wageningen The Netherlands; ^4^ Group for Population and Evolutionary Ecology, FB 02, Institute of Ecology University of Bremen Leobener Str. 5 28359 Bremen Germany; ^5^ CABI Rue des Grillons 1 2800 Delémont Switzerland; ^6^ Animal Breeding and Genomics Wageningen University & Research PO Box 338 6700 AH Wageningen The Netherlands; ^7^ Institut de Recherche en Biologie Végétale Université de Montréal 4101 Sherbrooke Est Montréal Quebec Canada H1X 2B2; ^8^ Instituto Valenciano de Investigaciones Agrarias (IVIA), Centro de Protección Vegetal y Biotecnología Unidad Mixta Gestión Biotecnológica de Plagas UV‐IVIA Carretera CV‐315, Km 10'7 46113 Moncada Valencia Spain; ^9^ Laboratory of Entomology Wageningen University & Research Droevendaalsesteeg 1 6708 PB Wageningen The Netherlands; ^10^ Biosystematics Group Wageningen University & Research Droevendaalsesteeg 1 6708 PB Wageningen The Netherlands; ^11^ Department of Genetics, Estructura de Recerca Interdisciplinar en Biotecnología i Biomedicina (ERI‐BIOTECMED) Unidad Mixta Gestión Biotecnológica de Plagas UV‐IVIA, Universitat de València Dr Moliner 50 46100 Burjassot Valencia Spain; ^12^ Koppert Biological Systems Veilingweg 14 2651 BE Berkel en Rodenrijs The Netherlands; ^13^ Department of Biochemistry and Biotechnology University of Thessaly Biopolis 41500 Larissa Greece; ^14^ Institut für Populationsgenetik Vetmeduni Vienna Veterinärplatz 1 1210 Vienna Austria; ^15^ Vienna Graduate School of Population Genetics Vetmeduni Vienna Veterinärplatz 1 1210 Vienna Austria; ^16^ INRA, CNRS, UMR 1355‐7254 400 Route des Chappes BP 167 06903 Sophia Antipolis Cedex France; ^17^ Kearney Agricultural Research and Extension Center University of California Berkeley 9240 South Riverbend Avenue Parlier CA 93648 USA; ^18^ Netherlands Institute of Ecology (NIOO‐KNAW) Droevendaalsesteeg 10 6708 PB Wageningen The Netherlands; ^19^ Institute of Entomology Biology Centre CAS Branišovská 31 370 05 České Budějovice Czech Republic; ^20^ Faculty of Science University of South Bohemia Branišovská 1760 370 05 České Budějovice Czech Republic; ^21^ Department of Biology University of Rochester Rochester NY 14627 USA; ^22^ cE3c: Centre for Ecology, Evolution, and Environmental Changes Faculdade de Ciências da Universidade de Lisboa Edifício C2, Campo Grande 1749‐016 Lisbon Portugal

**Keywords:** artificial selection, biological control, genetics, genome assembly, genomics, insect breeding, microbiome, modelling

## Abstract

Biological control is widely successful at controlling pests, but effective biocontrol agents are now more difficult to import from countries of origin due to more restrictive international trade laws (the Nagoya Protocol). Coupled with increasing demand, the efficacy of existing and new biocontrol agents needs to be improved with genetic and genomic approaches. Although they have been underutilised in the past, application of genetic and genomic techniques is becoming more feasible from both technological and economic perspectives. We review current methods and provide a framework for using them. First, it is necessary to identify which biocontrol trait to select and in what direction. Next, the genes or markers linked to these traits need be determined, including how to implement this information into a selective breeding program. Choosing a trait can be assisted by modelling to account for the proper agro‐ecological context, and by knowing which traits have sufficiently high heritability values. We provide guidelines for designing genomic strategies in biocontrol programs, which depend on the organism, budget, and desired objective. Genomic approaches start with genome sequencing and assembly. We provide a guide for deciding the most successful sequencing strategy for biocontrol agents. Gene discovery involves quantitative trait loci analyses, transcriptomic and proteomic studies, and gene editing. Improving biocontrol practices includes marker‐assisted selection, genomic selection and microbiome manipulation of biocontrol agents, and monitoring for genetic variation during rearing and post‐release. We conclude by identifying the most promising applications of genetic and genomic methods to improve biological control efficacy.

## I. INTRODUCTION

Biological control (the use of natural enemies to control pests), is arguably the best solution for phasing out large‐scale pesticide use (Thomas & Willis, 1998; Bale, van Lenteren, & Bigler, 2008). It has been broadly applied for hundreds of years and to great success in both greenhouse and open field systems worldwide (van den Bosch, 1971; Stiling & Cornelissen, 2005). Most research into the fundamentals of biological control has been from an ecological perspective, focusing on aspects such as optimal foraging and risk monitoring (Wajnberg, Bernstein, & van Alphen, 2008; Heimpel & Mills, 2017). As clearly not all programs result in the desired level of pest management, there is considerable room for improvement (Wajnberg, 2004). The reasons for biocontrol programs not always reaching their full potential are manifold, ranging from releasing the wrong control agents, agents not being adapted to local conditions, undesired interactions with the native fauna, and evolutionary changes in the pest species upon invasion.

**Fig 1:**
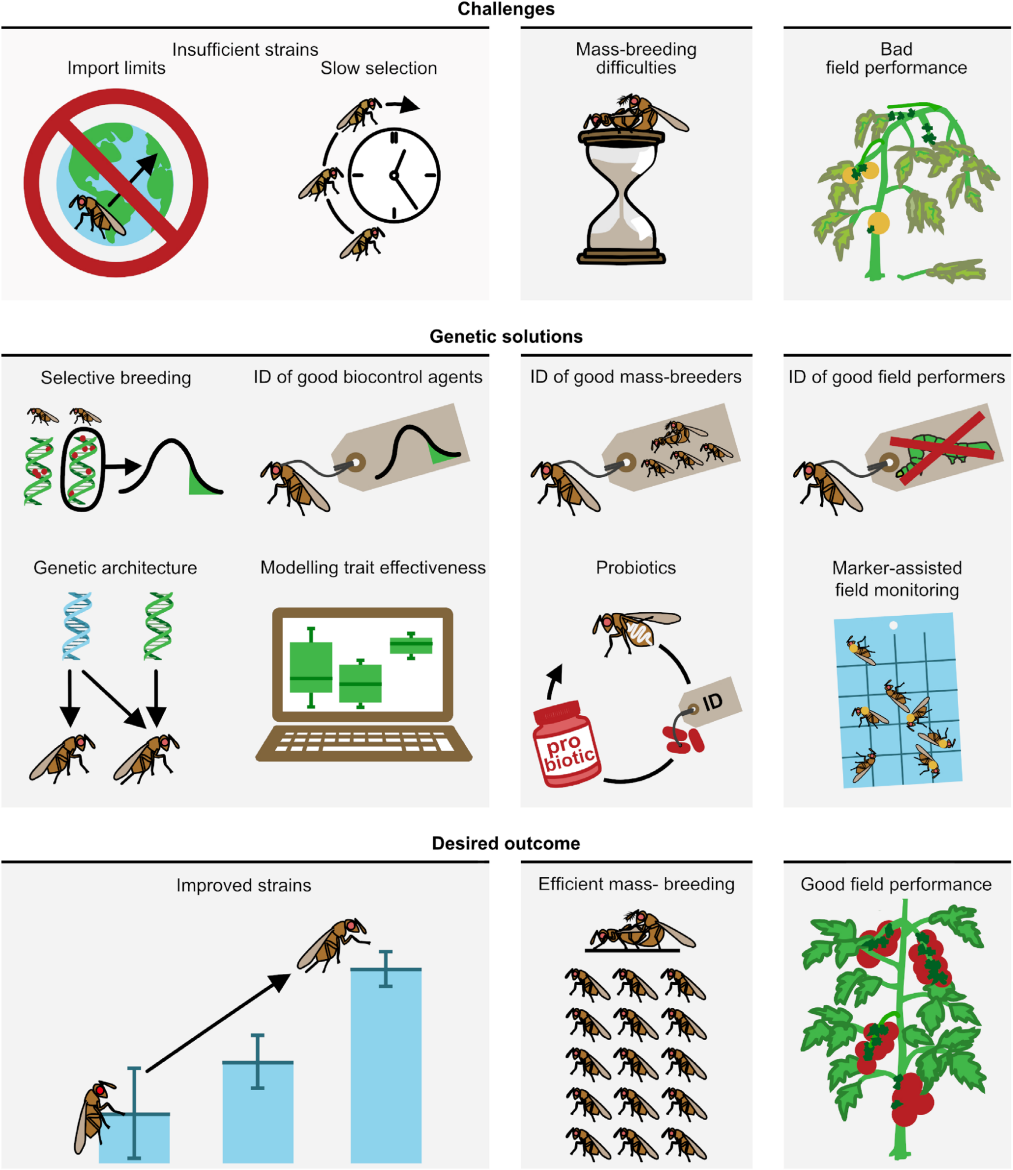
Overview of the potential of genetic methods to address biocontrol challenges.

In the past, the default method for improving biocontrol was to find a more efficient wild species or strain as the biocontrol agent (Hassan & Guo, 1991; Hassan, 1994; Nomikou *et al*., 2001; Hoelmer & Kirk, 2005). However, the Nagoya protocol for Access and Benefit Sharing of Genetics Resources has severely limited international exchange of biological materials, so sourcing more‐effective biocontrol agents from the field has become severely restricted (Cock *et al*., 2010; Deplazes‐Zemp *et al*., 2018; Mason *et al*., 2018). Moreover, certain geographical regions strictly regulate which agents can be used, e.g. only local strains originating from the region itself (Loomans, 2007; Hunt, Loomans, & Kuhlmann, 2011). Concurrently, demand for more effective biocontrol agents is rising, driven by the growth of the organic food production market (valued at 62.9 billion USD as of 2013; Willer & Lernoud, 2019; Baker, Green, & Loker, 2020). The global biological control market was worth 1.7 billion USD in 2015, with sales growing three times faster than pesticides (van Lenteren *et al*., 2018). Additionally, policy developments have aimed to reduce synthetic pesticide use (van Lenteren *et al*., 2018) such as an EU‐wide neonicotinoid ban (Gross, 2013; Stokstad, 2018) and continuous curtailment of organophosphate use in the USA and worldwide (Hertz‐Picciotto *et al*., 2018). The rise of the organic market in conjunction with a reduction of pesticide use has resulted in the rapid growth and increased market value of the biocontrol industry (de Clercq, Mason, & Babendreier, 2011; Dunham, 2015; van Lenteren *et al*., 2018). It is now more urgent than ever to understand how to improve effectively and efficiently non‐native biocontrol agents already in use and to develop novel native biocontrol agents. We review recent developments in the field of biological control that indicate that genetics‐based solutions are key (Fig. 1).

For decades, genetic methods have been advocated to improve the efficacy of biocontrol programs (White, Debach, & Garber, 1970; Hoy, 1986; Hopper *et al*., 1993; Narang, Bartlett, & Faust, 1993; Nunney, 2003; Routray *et al*., 2016; Kruitwagen, Beukeboom, & Wertheim, 2018; Lirakis & Magalhães, 2019). Significant genetic variation has been demonstrated in several key life‐history and behavioural traits of potential biocontrol agents (Hoy, 1985; Rousch, 1990; Hopper *et al*., 1993; Wajnberg, 2004; Ferguson, 2020). However, despite its proven application in crop and livestock breeding, there have been few attempts to improve biocontrol agents through genetic means. We currently witness a revival of the idea to apply genetics to the improvement of biocontrol programs, by exploiting intra‐ and interspecific variation (Lommen, de Jong, & Pannebakker, 2017; Kruitwagen *et al*., 2018), performing experimental evolution (Lirakis & Magalhães, 2019), adapting the microbiome for improved rearing methods (Ras *et al*., 2017; Koskinioti *et al*., 2020a, 2020b), and by population and field‐monitoring of released agents (Roderick & Navajas, 2003; Stouthamer & Nunney, 2014; Coelho *et al*., 2016). Notably, these methods all employ classic genetics principles within a species’ existing gene pool, distinguishing them from genetically modified organisms (GMOs) that have DNA from foreign organisms introduced into their genomes. That means that the methods discussed here comply with the requirements of the organic food industry and any policies that limit or prohibit GMO use (Gomiero, Pimentel, & Paoletti, 2011). Next to genetic applications, we need an evolutionary perspective on the sustainability and risks of biocontrol programs, for example to enable predictions of future adaptations of pests and agents (Hufbauer & Roderick, 2005; Szűcs *et al*., 2019). Finally, application of genetics and genomics in biocontrol programs cannot be evaluated without considering the role of environmental and ecological processes (Thrall *et al*., 2011).

**Fig 2:**
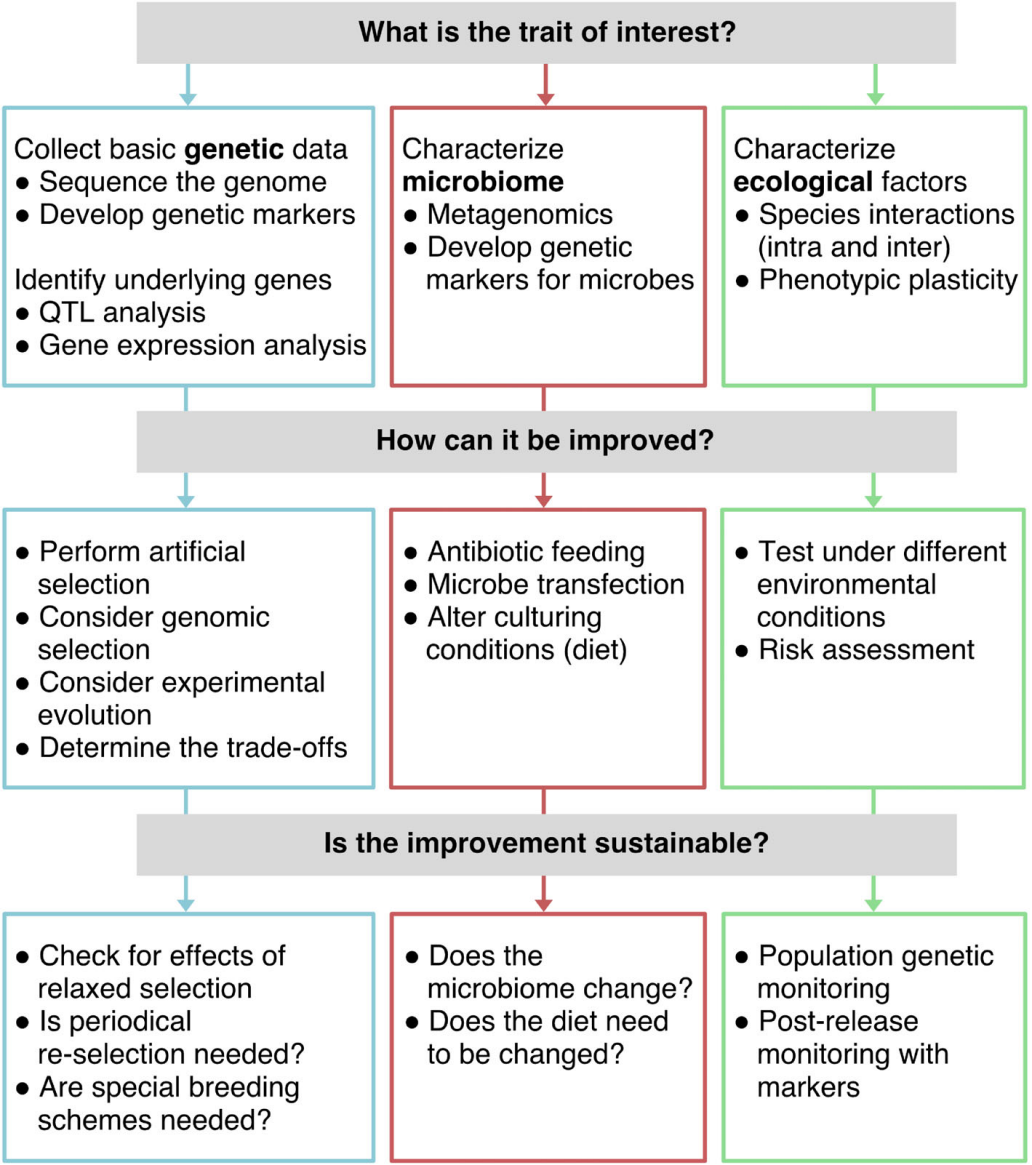
Guide to the use of genetic methods in research and development, sorted according to research question. QTL, quantitative trait locus.

We first identify which organismal traits are important for biological control and should therefore be targeted for improvement (so‐called ‘biocontrol traits’). Next, we present the current state and future prospects of using genetic and genomic methods towards that aim. We consider these methods from evolutionary and ecological contexts, that is how these methods can realistically operate in long‐term breeding programs and in the field (Fig. 2). Because of their prevalence and economic importance (van Lenteren *et al*., 2018), we focus on programs using arthropod biocontrol agents, although these universal genetics principles overlap with other agents (e.g. nematodes, fungi, bacteria). Although the most common form of biological control is augmentative (the recurrent release of a biocontrol agent population not expected to establish permanently, or to supplement an existing population), the methods discussed here can also be applied to classical biological control (release of a new agent with the intention of establishing a self‐sustaining population and level of pest control in the area of the pest) and conservation biological control (conserving natural habitat to increase populations of natural enemies). As genomics are key to many of these methods, we provide a key on how to obtain genome‐based resources in specific biocontrol contexts. We conclude by reviewing present uses and forecasting applications of the most promising genetic and genomic methods in the future.

## II. WHAT ARE BIOCONTROL TRAITS?

One of the prime reasons preventing the uptake of genetic improvements of biocontrol agents is difficulty in deciding which traits to optimize. Candidate traits can be roughly subdivided into pest‐suppression ability, adaptation to abiotic factors, reducing ecological risk, and improving mass production or storage [see Kruitwagen *et al*. (2018) and Bielza *et al*. (2020) for a comprehensive overview]. For some traits, such as pest kill‐rate, the direction of improvement is apparent as killing more pests is a primary determinant of biocontrol success (Stiling & Cornelissen, 2005). However, for other traits, the direction to take is less obvious. For example, most biocontrol agents attack hosts/prey that are clumped in patches in the environment. Would it be more effective for the agent to clear patches completely before moving on, or to disperse rapidly to protect a larger total crop area (Wajnberg, Roitberg, & Boivin, 2016; Plouvier & Wajnberg, 2018)? The optimal strategy depends on the specific ecological circumstances faced by the biocontrol agent and the economic harm inflicted by pest species at low density. Optimality models based on behavioural ecology can provide important insights into the critical characteristics of natural enemies for successful biological control (Mills & Kean, 2010; Wajnberg *et al*., 2016; Lommen *et al*., 2017). Recently, Plouvier & Wajnberg (2018) developed a general modelling framework that identifies key biocontrol agent life‐history traits from an economical perspective. For dispersal‐and‐predation simulations, two different optimized life‐history strategies for the agents were found to result in higher potential economic returns, differing in plant‐leaving decision and host handling time of the biocontrol agent, but also in their respective fecundity, longevity, and dispersal ability. Such a general modelling framework can be parameterised for biocontrol species with different biology (including parasitoids and predators) and different ecological situations to help identify the key traits to target for genetic improvement.

A key requirement for biocontrol traits to be targeted for selection is significant heritability (the proportion of the total phenotypic variation among individuals that is due to additive genetic variation), which allows a trait to be improved by (artificial) selection. The amount of standing genetic variation depends on the type of trait and its genetic architecture, which can be defined as the number and location of the loci involved, and their interactions (such as dominance, epistasis and pleiotropy). Traits closely associated with fitness that are important for biocontrol, such as life‐history and behavioural traits (Mousseau & Roff, 1987; Wajnberg, 2004; Lommen *et al*., 2017; Kruitwagen *et al*., 2018; Xia *et al*., 2020), typically have lower heritabilities than physiological and morphological traits (Mousseau & Roff, 1987). The amount of genetic variation for biocontrol traits is currently poorly investigated and insufficiently known. For example, in a recent review of arthropod biocontrol literature, of 2927 articles identified as investigating genetics of biocontrol traits, only 69 reported data on genetic variation (Ferguson, 2020). Selection of low‐heritability traits towards optimal values is possible, but the efficiency of this process depends on a trait's genetic architecture. It is therefore of key importance to uncover the genetic architecture of biocontrol traits if we aim efficiently to improve them.

## III. WHAT GENETIC INFORMATION DO WE NEED?

### (1) Genome assembly

Assembling a genome for a biocontrol agent of interest vastly expands the possibilities for generating new knowledge on the genetic architecture of biocontrol traits. A reference genome facilitates studies that focus on gene expression analyses, targeted gene editing, and marker‐informed selection. Although producing a high‐quality genome (high coverage, few gaps) is often portrayed as an essential goal (Bentley, 2006; Faino & Thomma, 2014), a high‐level resolved genome may often not be required. Instead, sequences may be collected, assembled, and annotated to the level required for a specific project, and the genome can later be improved to the level desired by other parties (Papanicolaou *et al*., 2017). In other words, in more applied circumstances, such as biological control, the aim may be a ‘good‐enough’ genome rather than a high‐quality genome. Also, some applications can already be realised with an incomplete genome, including the quick generation of molecular markers such as microsatellites (Grbić *et al*., 2011; Abe & Pannebakker, 2017; Kamimura *et al*., 2019) for low‐cost analysis of genetic variation (Baker, Loxdale, & Edwards, 2003; Paspati *et al*., 2019) and linkage map construction (Beukeboom *et al*., 2010; Niehuis *et al*., 2010).

**Fig 3:**
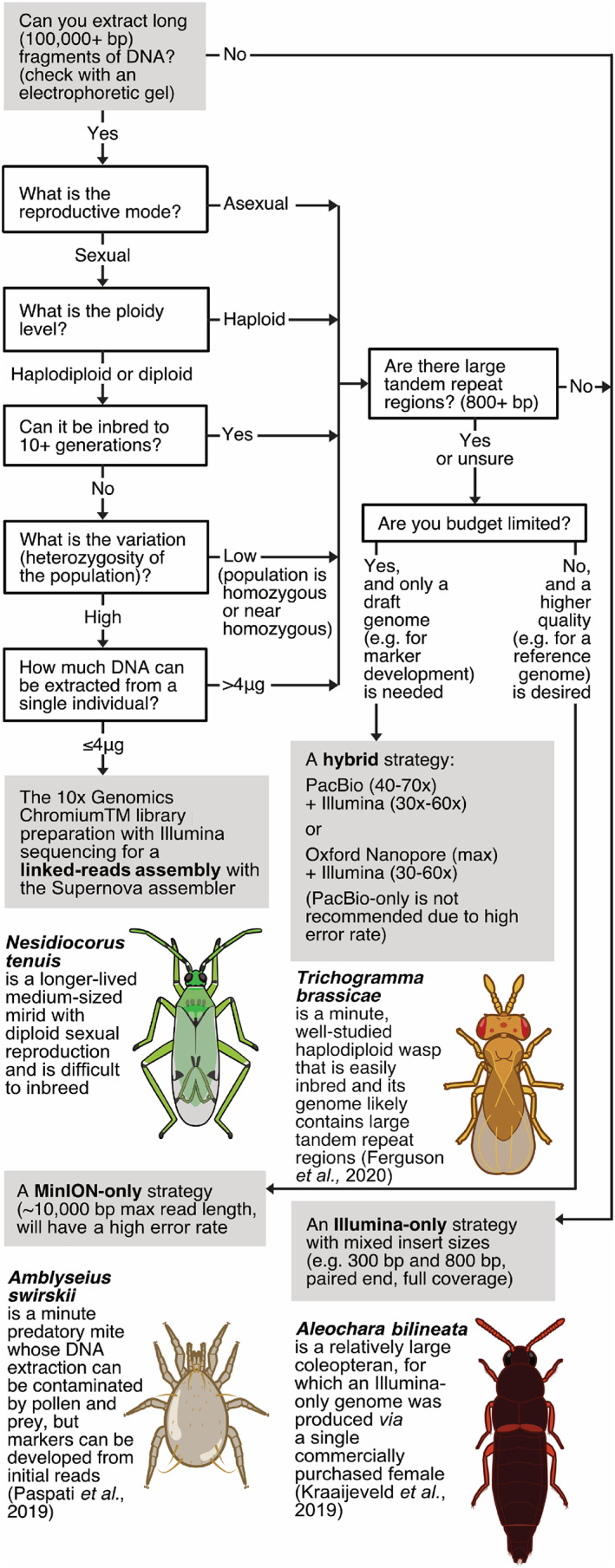
Sequencing strategy key for obtaining genomes of biocontrol agents with selected examples of species. bp, base pair.

Genome assembly goes through various stages: sequencing from an inbred stock or a single individual, aligning the sequences into an assembly, and annotating the assembly with protein‐coding information (Ekblom & Wolf, 2014). Although still requiring a considerable amount of labour and funding, recent technological advances have lowered the cost of sequencing a genome considerably (Wetterstrand, 2019). In the context of biological control, successfully producing a workable genome within one's budget and objectives requires careful strategy. For example, for many biocontrol agent species, the amount of DNA extracted from a single individual (because of small body size) is insufficient for sequencing a genome (Richards & Murali, 2015; Cruaud *et al*., 2019). Pooling many genetically identical individuals is a solution, but how to obtain such a sample varies among species; this is easier and has been done, for instance, with isofemale lines of haplodiploid parasitoids (Werren *et al*., 2010; Geib *et al.,* 2017; Ferguson *et al*., 2020) but can be more challenging for species that are difficult to inbreed (e.g. ladybirds; Facon *et al*., 2011). Figure 3 presents a key for deciding which sequencing strategy to use for various biological control situations, accounting for the current state of the technology, the biology of the species, and the objective for assembling the genome.

For relatively large‐sized species, such as the parasitoid beetle *Aleochara bilineata* Gyllenhal, a tissue sample from a single individual female was adequate for producing a high‐quality reference Illumina‐based genome; this was done *via* a third party (Kraaijeveld *et al*., 2019). By contrast, a single individual of the minute predatory mite *Amblyseius swirskii* Athias‐Henriot *swirskii* is inadequate for extracting enough DNA to sequence a genome. A solution was found by pooling enough genetically identical individuals to meet the initial genomic weight requirement, but this required an additional, logistically difficult inbreeding step (Paspati *et al*., 2019) that was not necessary for *A. bilineata*. A draft genome was created for *A. swirskii* with inbred individuals using the laboratory benchtop sequencer MinION. This genome was not intended to serve as a high‐quality, long‐term reference genome for this species, but was used to develop markers affordably for tracking genetic diversity in mass‐reared populations (Paspati *et al*., 2019). This approach had the advantage of flexible in‐house troubleshooting with an affordable machine and materials.

### (2) Gene discovery

Mapping genes has a long tradition in breeding and research, particularly using quantitative trait loci studies (QTL) [e.g. maize height (Burr *et al*., 1988), soybean seed morphology (Mansur *et al*., 1993), pig fatness and growth (Andersson *et al*., 1994) and *Nasonia* parasitoid wasp sex ratio (Pannebakker *et al*., 2011)]. A QTL study uses crosses of individuals with different extreme phenotypes and links their segregation in offspring to molecular marker data to identify the genetic basis of complex traits (Lynch & Walsh, 1998; Beukeboom & Zwaan, 2007). High‐throughput sequencing and genome‐wide association studies (GWAS) have enabled higher resolution mapping screens (Schlötterer *et al*., 2014), that is identification of loci with different allele frequencies between two populations with different phenotypes of the target trait (Bastide *et al*., 2013).

For QTL mapping and GWAS, the statistical power to identify causative variants increases with the number of individuals analysed. In addition, in a QTL approach, power increases with the number of generations invested, but mapping precision is typically lower than GWAS. The genotyping costs can be reduced by relying on sequencing pools of individuals with extreme phenotypes (Pool‐GWAS; Schlötterer *et al*., 2014). This approach was used to create a genome‐wide map for body pigmentation in *Drosophila melanogaster* Meigen (Bastide *et al*., 2013) and can theoretically be applied to any target trait in any arthropod. Although individuals for these studies can be sourced from commercial biocontrol populations, these tend to be inbred and contain only a fraction of the genetic variation harboured by natural populations (Rasmussen *et al*., 2018; Paspati *et al*., 2019). For exploratory studies, sampling from wild populations may be better for collecting sufficiently variable individuals with clear segregation of phenotype, and thus correspondingly distinct genotypes for candidate loci. Alternatively, as it may be legally or logistically difficult to sample multiple natural populations across a large geographic range, commercial strains that are already in use and have contrasting phenotypes may be used instead. For instance, long‐established *Trichogramma cacoeciae* Marchal strains originating from France and Tunisia have higher fecundity under different temperatures (Pizzol *et al*., 2010), and would be good candidates for investigating loci linked to climate adaptation.

Another approach to delineating the genomic architecture of biocontrol traits is studying gene expression. Sequencing transcriptomes and proteomes, which are complete RNA and protein expression profiles of an organism respectively, has become increasingly easy and affordable. The advantage of gene expression studies is that they delve into context‐dependent phenotypes. For example, gene expression differences between the sexes are highly relevant for parasitoid wasps (Wang, Werren, & Clark, 2015) because females have active genes for host‐feeding, envenomation and oviposition that males lack. Spatiotemporal expression pattern differences of protein and transcript quantity, methylation, RNA splicing, and post‐translational modification may be responsible for sex‐specific phenotypes, and can be used to find trait‐linked loci even when genetic sequences are identical between males and females (Wang *et al*., 2015). Such studies have been used to delineate the architecture of, for example, sex determination (Verhulst, Beukeboom, & van de Zande, 2010), oviposition (Pannebakker *et al*., 2013; Cook *et al*., 2015), and venom composition (de Graaf *et al*., 2010) in *Nasonia vitripennis* (Walker), and antennal perception of different olfactory cues, i.e. male mate‐searching *versus* female host‐searching in the parasitoids *Cotesia vestalis* (Haliday) (Nishimura *et al*., 2012) and *Chouioia cunea* Yang (Zhao *et al*., 2016).

Analysis of transcriptomic and proteomic data obtained at different environmental or culturing conditions is also a powerful tool, as it can both identify and quantify patterns of gene expression (Wang *et al*., 2009). For example, transcriptome analyses of the ladybird *Cryptolaemus montrouzieri* Mulsant or the parasitic wasps *Cotesia typhae* Fernández‐Triana and *Lysiphlebus fabarum* Marshall show that these biocontrol agents have adapted to alternative prey/hosts by modifying the regulation of genes mainly related to development, digestion, detoxification and virulence (Li *et al*., 2016; Benoist *et al*., 2017; Dennis *et al*., 2017). Mechanisms underlying resistance to certain pesticides in the predatory mite *Neoseiulus barkeri* Hughes and the ladybird beetle *Propylaea japonica* (Thunberg) were identified by analysing RNA sequencing (RNAseq) data (Tang *et al*., 2014; Cong *et al*., 2016). Transcriptomics may also pave the way to understanding symbiont‐mediated resistance to parasitism (Oliver, Moran, & Hunter, 2005), and help to reverse this effect or to make parasitoids more virulent. Additionally, proteomic analysis of aphid parasitoids *Aphidius colemani* Haliday that were either exposed to fluctuating high and low temperatures or to constant cold provided insight on genes and proteins involved in surviving temperature extremes, such as those involved in energy metabolism (Colinet *et al*., 2007). Nutrigenomics (how diet affects gene expression) also has great potential for improving artificial diets for mass rearing of biocontrol agents, and for selecting strains that develop particularly well on such diets (Coudron, Yocum, & Brandt, 2006; Yocum, Coudron, & Brandt, 2006). A final interesting application of transcriptomics for biocontrol is to identify the genetic architecture of memory and learning, as parasitoids can be trained to recognise host species (Huigens *et al*., 2009). Recently, genes in the Ras (rat sarcoma) and phosphoinositide 3‐kinase (PI3K) pathways were found to be responsible for interspecific differences in *Nasonia* memory retention (Hoedjes *et al*., 2014, 2015).

Gene‐expression studies can thus contribute to understanding adaptation mechanisms of biocontrol agents to a new environment, prey/host defences or novel hosts. Yet careful control of expression data collection, such as consistent life stage or common garden conditions, is important, as phenotypic plasticity can add noise to analyses. Ultimately, understanding gene expression patterns is essential to allow the preservation of a robust phenotype, or how likely a phenotype is to persist in various agro‐ecological environments (Félix & Barkoulas, 2015).

### (3) Genome editing for exploratory research

Advances in genomics approaches and knowledge have made it possible to modify certain regions in the genome of an organism to study how such modifications are reflected in its phenotype. New phenotypic variants can be generated by knocking‐down or knocking‐out genes. Knocking‐down refers to temporary gene expression inhibition through RNA interference (Pratt & MacRae, 2009). Knocking‐out refers to permanent alteration through the germ line, and the most advanced of these knock‐out approaches is clustered regulatory interspaced short palindromic repeats (CRISPR) (Hsu, Lander, & Zhang, 2014). Knocking‐down or knocking‐out candidate biocontrol trait genes can lead to insights regarding their functions that can be used to optimize selection or breeding of biocontrol agents. For example, they can be used to examine the role of genes in a trait through linkage with the null phenotypes, and those genes can be specifically targeted for selection. Currently, gene‐editing technology is exploratory and for fundamental research use, but should not be used in novel biocontrol release programs. Although no external DNA is introduced with knock‐down or knock‐out, some countries consider gene‐editing techniques to be in the same legal category as GMOs (EU; Callaway, 2018) whereas others do not [USA (Kim & Kim, 2016; Waltz, 2016); Australia (Mallapaty, 2019)]. They are therefore subject to many of the same regulations that vary broadly in restrictiveness, e.g. allowed in the USA *versus* a complete ban in the EU (reviewed in Alphey & Bonsall, 2018). In addition, the compatibility of gene‐editing with the current ‘non‐GMO’ marketability of biological control is questionable.

### (4) Microbiomes

Currently, there is much interest in the role of the microbiome in organismal functioning. In a biocontrol context, it is known that microbes can generate chemical signals that attract parasitoids to their host, and that bacteria can have a defensive role against parasitoids, such as in aphids (Oliver, Moran, & Hunter, 2006; Schmid *et al*., 2012; Rothacher, Ferrer‐Suay, & Vorburger, 2016; Jamin & Vorburger, 2019; Koskinioti *et al*., 2019; Dicke, Cusumano, & Poelman, 2020). Nowadays, universal DNA markers can be applied to characterise the microbiome, that is to identify all bacterial symbionts to at least family or genus level, and their proportionate presence (Ras *et al*., 2017). This can be used to infer the relative abundance and relative contribution of each symbiont to biocontrol traits. However, despite the enormous attention on the role of microbiomes, we still know very little about whether and how microbes contribute to arthropod life‐history traits and biocontrol traits in particular (Janson *et al*., 2008; Brinker *et al*., 2019; Gurung, Wertheim, & Falcao Salles, 2019). In addition, the factors that determine the microbiome composition are often not well known. Such information is important to judge how consistent the microbiome is transgenerationally, and if it can be manipulated through rearing.

## IV. HOW CAN GENETICS BE USED TO IMPROVE BIOLOGICAL CONTROL?

### (1) Artificial selection

The traditional approach to improving biocontrol agents has been through artificial selection. Artificial selection exploits inter‐individual genetic variation of life‐history or behavioural traits. For example, agent species have been selected to improve tolerance to climatic conditions to expand their geographic range of use (White *et al*., 1970), pesticide resistance (allowing their compatibility with pesticide spraying) (Roush & Hoy, 1981*b*; Spollen & Hoy, 1992), plant adaptation, especially to tomato in strains of *Phytoseiulus persimilis* Athias‐Henriot (Drukker *et al*., 1997) and development time, to speed up production (Rodriguez‐Saona & Miller, 1995). Rather than performing crosses of individuals with desired traits, experimental evolution exposes populations to specific environmental conditions for several generations and determines the effect on the trait of interest. Although generally successful (Lirakis & Magalhães, 2019), artificial selection remains underutilised in biological control (Wajnberg, 2004; Lommen *et al*., 2017; Kruitwagen *et al*., 2018). There are several reasons for this. One obvious reason is lack of sufficient genetic variation because too few individuals are collected for a source population (but little is known about such unsuccessful attempts in the literature). A second possibility is that once selected, the genetic variation in the selected population will change through stochastic processes such as genetic drift (Roush & Daly, 1990; Stouthamer, Luck, & Werren, 1992; Hopper *et al*., 1993; Wajnberg, 2004). The conclusion has been that to avoid these issues arising from ‘selection relaxation’, biocontrol agents must be continuously re‐selected, which can be economically prohibitive. There is, however, little empirical evidence for this, and a recent study on *Drosophila* indicates that laboratory populations may not change much in life‐history parameters compared to their natural counterparts (Michalak *et al*., 2019).

Trade‐offs between life‐history traits are also a factor in biocontrol evolution. Life‐history theory poses that an organism has limited resources to allocate to each trait. To select for the enhancement of one or more traits, as is the goal in biocontrol breeding, pleiotropic and disadvantageous changes can occur in other traits, and the overall effect on the biocontrol function of the organism can be unpredictable (Stearns, 1989; Roff, 2007). A trade‐off may be detrimental for biocontrol because it affects negatively either another trait or another stage in the biocontrol program. For instance, it has been noted that traits corresponding to high yield and ease of use under laboratory and industrial conditions are favourable, but adaptation to captivity may come at the expense of an agent's efficacy in the field (Mackauer, 1976; Hopper *et al*., 1993; Sørensen, Addison, & Terblanche, 2012; Sánchez‐Rosario *et al*., 2017). An example is laboratory maintenance negatively impacting female fecundity, body size, host‐killing, and host‐searching in the parasitoid *Muscidifurax raptor* Girault and Sanders (Geden *et al*., 1992). It is also logistically difficult to phenotype complex traits for many biocontrol agents (e.g. measuring total fecundity, total host‐killing, dispersal ability, etc.).

However, the means to address many of these problems are at hand. There is evidence, for example, that selection relaxation may not be as problematic as previously believed. Theoretically, if the selection regime is strong enough, the trait goes to fixation in a population, i.e. the entire population will carry the desired trait and it is no longer subject to drift (Falconer & Mackay, 1996). Moreover, several traits that are selected apparently entail no trade‐off in other traits. This is reflected in the value of selected traits being maintained in insect lines even after many generations of no active selection on the trait (e.g. White *et al*., 1970; Croft & Meyer, 1973; Roush & Hoy, 1980; see Lirakis & Magalhães, 2019).

**Fig 4:**
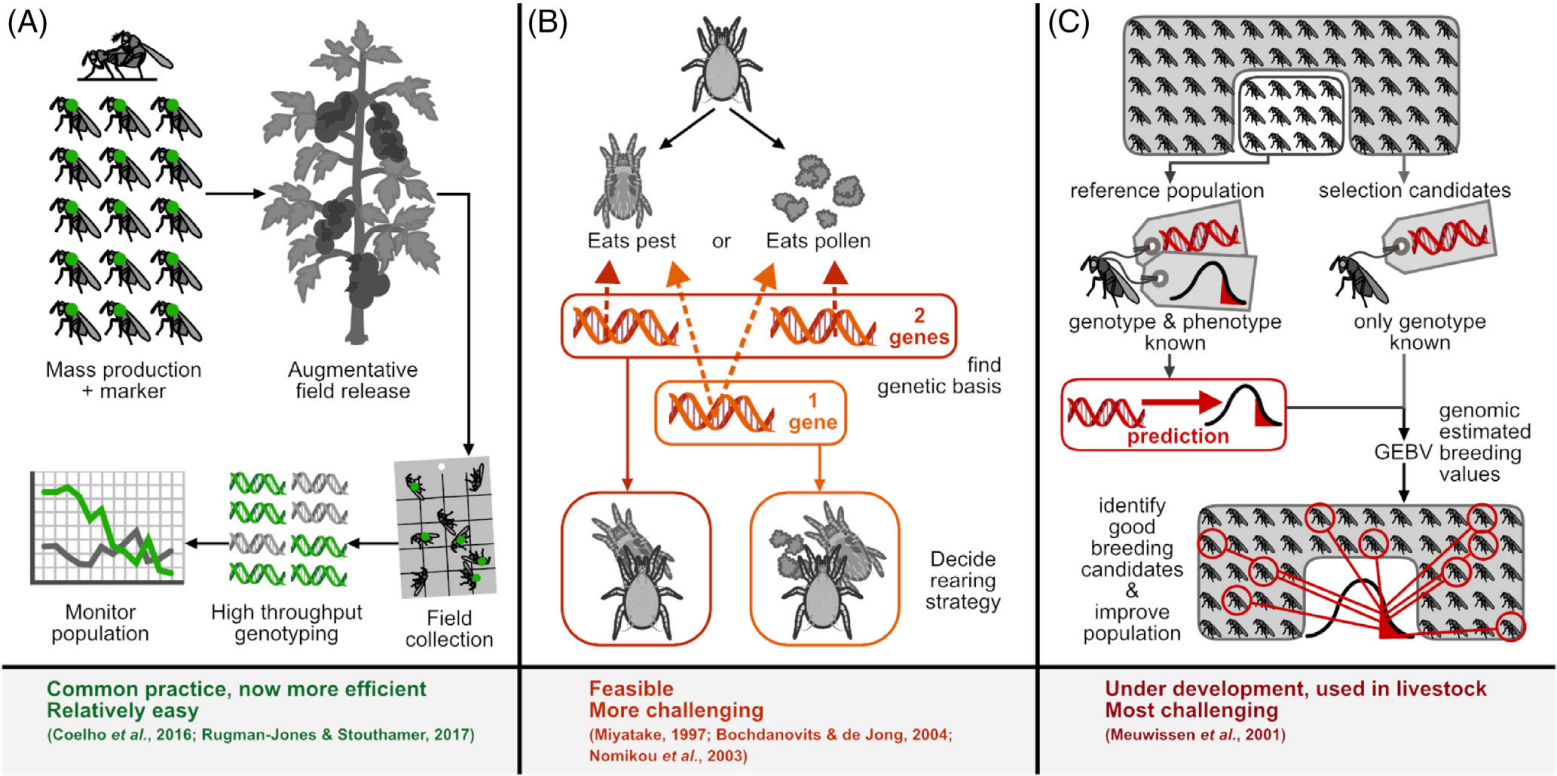
Examples of the application of genetic techniques in biocontrol, in increasing order of complexity. (A) Genotyping for field monitoring of released biocontrol agents. (B) Design of optimal rearing strategy based on genetic architecture of traits of interest. (C) Genomic selection to improve polygenic or hard‐to‐phenotype traits.

Key to these solutions is understanding the genetic architecture of the trait under selection, which would allow the combination of individual phenotype selection with molecular genetics through applying marker‐assisted selection (Lande & Thompson, 1990). When, for instance, we know the loci and alleles associated with a desired or undesired target trait, we can breed a more efficient biocontrol agent by selecting for the former and avoiding the latter. Not all trade‐offs may be detrimental to biological control. For example early reproduction may come at a cost of longevity (Williams, 1996), but a long life may not be important to a captive population's net productivity if it is frequently supplemented with new, fecund individuals. Knowing the genetic underpinning of trade‐offs between traits would assist in understanding and preventing unwanted correlated responses to selection (Fig. 4B). For example, antagonistic pleiotropy (genes operating on multiple traits but in opposing directions) is known for fecundity *versus* longevity in the melon fly, *Zeugodacus cucurbitae* (Coquillett) (Miyatake, 1997) and higher larval survival *versus* lower adult body mass in *D. melanogaster* (Bochdanovits & de Jong, 2004); additional studies could uncover these in biocontrol agent species. If there are variable pathways corresponding to different trade‐offs, it should be possible to select through one with the fewest unfavourable trade‐offs, exploiting the ubiquitous presence of genetic redundancy.

Learning behaviour is another biocontrol trait that responds to selection. Parasitoids (Dukas, 2000; van den Berg *et al*., 2011; Hoedjes *et al*., 2014, 2015; Kraaijeveld *et al*., 2018; Kruidhof *et al*., 2019), and predators (Rahmani *et al*., 2009; Schausberger *et al*., 2010) can be directly selected to recognise pest species better, or indirectly to recognise more efficiently recruitment signals from plants following herbivore attack (tritrophic interactions) (van der Putten *et al*., 2001; Turlings & Wäckers, 2004). Juvenile predatory *Phytoseiulus persimilis* Athias‐Henriot can be trained to accept specific prey species and retain this habit throughout its lifetime (Rahmani *et al*., 2009), as can numerous parasitoid wasps (reviewed in Kruidhof *et al*., 2019). Delineating the genetic architecture of memory and learning can thus assist in breeding lines that can learn and retain this information better.

Knowledge of the genetic architecture of traits can assist with artificial selection through deliberate targeting of linked genes. This is particularly helpful for traits that are laborious or challenging to phenotype repeatedly. For example, for many arthropods, assaying lifetime reproductive output would require counting thousands of offspring and waiting until the animal dies. In the meantime, work is invested in caring for the next generation whether or not their progenitors prove to have high lifetime fecundity. Instead, rather than the trait itself, selection can target a linked molecular marker, which is called marker‐assisted selection (MAS) (Lande & Thompson, 1990). Identification of candidate genes and QTL through the aforementioned gene discovery methods are key to developing direct markers for genes that control the trait of interest, or markers that are in linkage disequilibrium with the trait and are proximate to the coding gene. Although much work remains to uncover the genetic bases of traits, there are already good results documented for arthropods linking genes and QTL to foraging (Page *et al*., 2000), grooming (Oxley, Spivak, & Oldroyd, 2010), and *Varroa* mite resistance in honey bees (Behrens *et al*., 2011); fertility in the parasitoid *Leptopilina clavipes* Hartig (Pannebakker *et al*., 2004); and sex ratio (Pannebakker *et al*., 2011), memory retention and olfaction (Hoedjes *et al*., 2014, 2015), host specificity (Desjardins *et al*., 2010), and pupal diapause (Paolucci *et al*., 2016) in *N. vitripennis*.

### (2) Genomic selection

For complex traits with highly polygenic bases or genes with complicated epigenetic effects, direct and linkage equilibrium MAS may not be possible. In such cases, it is possible to use markers that are linked to a total breeding value instead of any specific phenotype (reviewed in Dekkers, 2004). Genomic selection employs this concept potentially to circumvent the need for proving marker causality. The statistical method of genomic selection uses information from genome‐wide DNA‐markers such as single nucleotide polymorphisms (SNPs) to select for complex traits (Meuwissen, Hayes, & Goddard, 2001) (Fig. 4C). This method is particularly helpful when artificial selection (marker assisted or not) is hampered by low heritability either through strong environmental noise and/or low levels of additive genetic variance. The genomic selection method considers markers distributed throughout the whole genome and estimates an effect of each marker, irrespective of the statistical significance of this effect. The total estimated genetic effect of an individual is the sum of the effects of all its markers as the genomic estimated breeding value (GEBV). By including effects of all markers, this method avoids missing a substantial portion of the genetic variance contributed by loci of minor effects, in contrast to methods that aim to identify the causal genes underlying traits. Although genomic selection still requires the collection of both genotypes and phenotypes, this work only needs to be done for the initial reference population and then at infrequent iterations as the predictive power of the reference population is gradually reduced over generations, given the potential evolution of the G matrix.

An advantage of genomic selection over traditional selection methods is that a higher accuracy of GEBVs can be achieved for traits of low heritability, and for traits that cannot be recorded on the selected candidate itself, but can be predicted through its genotype (Meuwissen *et al*., 2001). Over the last decade, genomic selection has proved its potential in animal breeding, that is dairy cattle (Hayes *et al*., 2009; Luan *et al*., 2009; VanRaden *et al*., 2009) and pigs (Lopes *et al*., 2017), but it has not yet been applied to biocontrol agents. Genomic selection methods may be particularly useful because GEBV can be estimated directly from the genotype, without the need for accurate pedigrees that are lacking for most biocontrol agents. One current challenge to genomic selection is the cost of large‐scale SNP panels, but these are already undergoing a rapid reduction in difficulty and expense. Also, collection of a sufficient amount of DNA may require sacrificing the selected candidate, but breeding of close relatives, such as offspring or full siblings, may offer a solution.

### (3) Field monitoring of genetic variation, performance, and ecological risk

The availability of genetic markers allows for population genetic analyses, either at a coarse scale such as in the case of microsatellite panels, or at a fine scale such as dense SNP panels based on assembled genomes. A powerful application of population genetics in biocontrol is monitoring of agents released into the field (Fig. 4A), allowing for the monitoring of their genetic variation, field performance, and ecological risk.

An important use of population genetics in biocontrol is the assessment of genetic variation. Loss of genetic variation is expected to occur in all captive populations, through inbreeding, selection, and random genetic drift, even when mass‐reared at high numbers (Mackauer, 1976). Although this does not always result in fitness or performance loss [see Hopper *et al*. (1993) and references therein; de Clercq, Vandewalle, & Tirry (1998); Facon *et al*. (2011)], experiments have shown severe effects of inbreeding depression and domestication on the reproductive performance of large captive populations of *Drosophila* (Woodworth *et al*., 2002) and a Chinese biocontrol population of the parasitoid *Binodoxys communis* Gahan (Gariepy, Boivin, & Brodeur, 2014). Specific care should be taken when culturing parasitoid wasps. As haplodiploid species, parasitoids are generally expected to suffer less from inbreeding depression for fitness traits, but in some species a loss of genetic variation for sex‐determination loci results in sterile males, and can lead to extinction of smaller populations (Stouthamer *et al*., 1992; Zayed & Packer, 2005; Hein, Poethke, & Dorn, 2009; Retamal *et al*., 2016; Zaviezo *et al*., 2018; Leung, van de Zande, & Beukeboom, 2019). These potentially large effects on fitness and performance make the monitoring of genetic variation a key part of mass‐culturing biocontrol agents. For those species with an assembled genome, whole‐genome genotype‐by‐sequencing (GBS) techniques (Baird *et al*., 2008) allow for fine‐scale population analyses by providing accurate allele frequency estimates to track evolution at a genomic scale and identify genomic regions under selection in contrasting ecological situations (Davey & Blaxter, 2010). This can also lead the way to unravelling the genetic architecture of relevant biocontrol traits.

A biocontrol agent's performance and ecological risk in the field can also be monitored *via* genetics methods. For example, for parasitoids, juvenile development often occurs within the host, making them difficult to detect at this stage. Furthermore, many species are difficult to identify *via* morphology because they belong to mega‐diverse groups of minute insects that may not be phylogenetically well resolved (Stouthamer *et al*., 1999; Sumer *et al*., 2009; Cruaud *et al*., 2019). Dispersal, host specificity, and attack rate can all be determined with polymerase chain reaction (PCR) amplification to detect the presence of species‐specific DNA of parasitoids in hosts (Gariepy *et al*., 2007, 2008; Stahl *et al*., 2019). A similar approach has been taken for gut‐content analysis to measure predation of the target pest or screen for consumption of non‐target species [mostly for predators, e.g. Hoogendoorn & Heimpel (2001), Thomas *et al*. (2013), Brown *et al*. (2014) and Nguyen *et al*. (2019); but see Rougerie *et al*. (2011) for a parasitoid example], as well as constructing complex food‐webs (Krehenwinkel *et al*., 2017). These methods can be used to modify biocontrol programmes, for example by selection of more efficacious agents with fewer non‐target effects.

Traditional neutral markers have been successfully used for performance monitoring of released strains [e.g. for the parasitoid *Trichogramma pretiosum* Riley, in which laboratory fecundity was correlated to field efficacy for 35 isofemale lines over 3 days (Coelho *et al*., 2016) and for the olive fly parasitoid *Psittalia lounbysuryi* (Silvestri) for ongoing monitoring of a biocontrol programme in southern France (Bon *et al*., 2008; Malausa *et al*., 2010)]. However, high‐density population genomic methods, such as GBS, allow for more detailed tracking of the introgression of the genetic material into previously released populations (Stouthamer & Nunney, 2014).

Despite the importance of tracking the fate of released agents and their associated alleles, there has been little effort invested in species‐identification assays or genotype‐based post‐release monitoring in the field, possibly because of its logistic difficulty (Blossey & Skinner, 1999; Coombs & McEvoy, 1999; but see Rugman‐Jones & Stouthamer, 2017). Typically, preserved individuals are brought back to the laboratory for DNA extraction, PCR amplification, and sequencing. A recent novel approach allows real‐time identification of a biocontrol agent in the field by loop‐mediated isothermal amplification (LAMP) (Lee, 2017). This is a low‐cost alternative to PCR that can be conducted in a single test tube and at a single temperature. In a biocontrol context, this method has been tested to detect parasitoidism of the Asian chestnut gall wasp *Dryocosmus kuriphilus* Yasumatsu by *Torymus sinensis* Kamijo (Colombari & Battisti, 2016), but it could facilitate all the field‐monitoring applications described.

### (4) Microbiome manipulation

Microbiomes may constitute an important target for modifying biocontrol agent performance. The composition of the microbial community of an organism can be altered through rearing conditions (e.g. a probiotic diet), *via* a breeding regime or by genetic manipulation (Grau, Vilcinskas, & Joop, 2017; Ras *et al*., 2017). For example, *D. melanogaster* fed a probiotic bacterium were less susceptible to infections of pathogenic bacteria (Blum *et al*., 2013). Sterile male performance was enhanced and *Pseudomonas* pathogen levels were reduced when the Mediterranean fruit fly *Ceratitis capitata* (Wiedemann) was fed bacterial supplements (Ami, Yuval, & Jurkevitch, 2010). Although these studies focused on flies used in the sterile insect technique (mass‐releasing sterile males to outcompete wild individuals), the same principles can be applied to biocontrol agents. Supplemented diets, including those with probiotics, can potentially directly improve the performance of mass‐reared parasitoids and predators (Nomikou *et al*., 2003, Ras *et al*., 2017; Koskinioti *et a* *l*., 2020a, 2020b), as can feeding on factitious hosts or prey with a specific microbiome indirectly.

It is also possible for host specimens to transmit their microbes to released biocontrol agents and influence their physiology and ecology (Schuler *et al*., 2013). In such cases, microbial screening with genetic markers would be useful for investigating microbiome shifts that are responsible for phenotype alterations. It is also known that microbes can be responsible for chemical signals that attract parasitoids to their host. This implies that biocontrol agents can potentially be trained using these host microbiomes to be more efficient at finding hosts. In the case of defensive microbes in pest species, exposure to such microbes during development may confer immunity in the next generation (Ras *et al*., 2017). These applications are, however, still theoretical and will require microbiome determination of specific biocontrol agent–pest pairs, identification of relevant symbionts, and development of methods to optimise their use.

A specific class of potentially useful symbionts are those that manipulate their hosts’ reproduction, such as *Wolbachia* (Dedeine *et al*., 2001; Vavre, Fouillet, & Fluery, 2003; Werren, Baldo, & Clark, 2008). *Wolbachia* can cause cytoplasmic incompatibility, which acts as a reproductive barrier among species or strains (Bourtzis *et al*., 1996; Fouillet *et al*., 2000; Gotoh, Noda, & Hong, 2003; Werren *et al*., 2008). However, it is possible to cure arthropods of *Wolbachia* with antibiotics, permitting interspecies hybridisation or inter‐strain reproduction (Breeuwer & Werren, 1995). This can, for example, be exploited to create strains that cannot interbreed with native congeners, reducing ecological risk [e.g. predators and parasitoids (Floate, Kyei‐Poku, & Coghlin, 2006; Machtelinckx *et al*., 2009)].

*Wolbachia* is also implicated in thelytokous reproduction (female parthenogenesis) in numerous insect species, such as the *Drosophila* parasitoids *Asobara* and *Leptopilina* (Breeuwer & Werren, 1995; Dedeine *et al*., 2001; Schidlo *et al*., 2002; Kremer *et al*., 2009), aphid parasitoids (Starý, 1999), and *Trichogramma* species (Stouthamer, Luck, & Hamilton, 1990; Stouthamer & Kazmer, 1994). Thelytoky is particularly significant for parasitoids in biocontrol because of its potential for increasing production of host‐killling females. In addition, parthenogenesis induction by *Wolbachia* can be used as a tool for advanced genotypic selection, which exploits the gamete duplication mechanism that underlies parthenogenesis induction by *Wolbachia* and allows for fast selection of beneficial gene combinations in parasitoids for biocontrol (Russell & Stouthamer, 2011). In species that do not carry *Wolbachia*, intentional infection (Yamashita & Takahashi, 2018) can potentially be used to alter reproduction and life‐history traits. Such transfection applications require careful testing, as *Wolbachia* phenotypes are not always the same between species (Veneti *et al*., 2012). Also, *Wolbachia* can reduce the relative number of other potentially beneficial symbiotic bacteria (Audsley, Ye, & McGraw, 2017; Ye *et al*., 2017) and conversely, other microbiota can outcompete *Wolbachia* (Kondo, Shimada, & Fukatsu, 2005; Goto, Anbutsu, & Fukatsu, 2006; Hughes, Rivero, & Rasgon, 2014; Rossi *et al*., 2015). These competition dynamics within microbiomes (Brinker *et al*., 2019; Gurung *et al*., 2019) are an important consideration when releasing manipulated strains into the field, as is the fact that new microbes introduced *via* hosts may become permanent fixtures in their ecosystem.

## V. CONCLUSIONS

1. It is a misconception that genetic solutions to biocontrol problems have been too complex to attempt, explaining the perceived ‘lack of progress’ in recent decades (Poppy & Powell, 2004; Lommen *et al*., 2017; Kruitwagen *et al*., 2018). Rather, the simpler approach of sourcing superior strains from nature has been more common, but this approach is now severely restricted. Despite their general applicability, animal breeding techniques have not been exploited to their full potential in the biocontrol field.
2. As biocontrol agents need to perform in a complex ecological environment, this makes it hard to decide which traits to optimise, but we are progressively gaining more knowledge on this, for instance by applying modelling frameworks (Plouvier & Wajnberg, 2018).
3. Several novel genetic and genomic approaches are at hand, yet each application requires proper contextualisation for a realistic projection of success. Marker‐based methods (such as field‐tracking and strain identification) are already being implemented (Fig. 4A). Others are not yet in use but are imminently possible, such as integrating knowledge of genetic architecture to develop more effective breeding programs (Fig. 4B). Still others, such as genomic selection, are currently largely in the theoretical realm. This may be due to novel technologies being prohibitively labour intensive or expensive, or still requiring troubleshooting. The rapid development of genomic sequencing techniques and the resulting cost reductions (Wetterstrand, 2019) will find applications in biocontrol as they have in other biological fields and applications (Handelsman, 2004; Hudson, 2008; Tautz, Ellegren, & Weigel, 2010; Ashley, 2016; Hohenlohe *et al*., 2018; Supple & Shapiro, 2018). Some applications are already successful, such as determining performance of released agents by characterising their diets. Furthermore, even in the more advanced applications, it is likely that a first success will lead to rapid embracement by the scientific community and industry, parallel to the development of the PCR technique and the human genome project. For example, a proof‐of‐principle study on genomic selection for a biocontrol agent would prove its feasibility even if cost and efficiency still need further optimisation (Xia, 2020; Xia *et al*., 2020) (Fig. 4C).
4. Novel methodology to uncover the genetic architecture of life‐history traits, in combination with increasing investment in research and development by biocontrol companies, will rapidly expand the knowledge base of the biocontrol field. Gene‐editing techniques are a useful research tool for delineating this genetic architecture, but as the current biocontrol market depends on a reputation of using more traditional genetics methods, now is not the time to use gene‐edited organisms in the field.
5. Knowledge about genetic variation can be used to design artificial selection programs, either by traditional selective breeding (White *et al*., 1970; Ram & Sharma, 1977; Voroshilov, 1979; Roush & Hoy, 1981*a*; Hoy, 1986; Rosenheim & Hoy, 1988; Spollen & Hoy, 1992; Zhang *et al*., 2018), or more sophisticated genomic selection (Xia, 2020; Xia *et al*., 2020) or experimental evolution approaches (Lirakis & Magalhães, 2019). As strong directional selection may also cause genetic impoverishment and increase vulnerability to pathogens and diseases, such approaches should always be designed to maintain sufficient genetic variation.
6. A caveat is that these approaches do have their limitations; for example, a founding population may lack sufficient genetic variation to select upon (Mackauer, 1976), and even best practices and intentions are not fool‐proof (e.g. selection for higher host specificity *versus* unpredicted host shifts in the field; Follett *et al*., 2000).
7. The aim of this review is to stimulate the application of genetic and genomic methodology in next‐generation biological control. We hope that it will lead to an increasing awareness of the potential of biocontrol agent breeding among scientists, the biocontrol industry, growers, and the general public.
